# Microneedling for the Treatment of Acne Scars: A Scoping Review of Therapeutic Protocols, Combination Therapies, and Current Evidence

**DOI:** 10.1111/jocd.71285

**Published:** 2026-11-11

**Authors:** Luca Castellano, Angela Capponi, Pier Paolo Rovatti, Elisa Campomagnani, Ilaria Maccari

**Affiliations:** ^1^ Campomats Srl Riccione Italy; ^2^ Studio Associato Medica Latina Latina Italy; ^3^ PPR Day Clinic Verona Italy

**Keywords:** atrophic acne scars, microneedling, percutaneous collagen induction, platelet‐rich plasma, scoping review

## Abstract

**Background/Objectives:**

Atrophic acne scarring is a complex condition with no definitive single treatment. Microneedling (MN) is a safe alternative to ablative procedures because it preserves the epidermis, minimizing the risk of post‐inflammatory hyperpigmentation. This scoping review maps the available literature on the technical parameters, multimodal protocols, and knowledge gaps associated with mechanical MN for acne scar revision.

**Methods:**

Following JBI and Preferred Reporting Items for Systematic Reviews and Meta‐Analyses extension for Scoping Reviews (PRISMA‐ScR) guidelines, a systematic search was conducted across PubMed and Scopus for English‐language articles published over the last decade. Both primary clinical research and secondary evidence syntheses evaluating manual and motorized mechanical MN were included.

**Results:**

A total of 85 studies met the inclusion criteria. Extracted data reveal a dichotomy between manual dermarollers and motorized pens, with penetration depths ranging from 0.5 to 3.0 mm. Because MN monotherapy shows limited efficacy for complex lesions, current protocols have shifted clearly toward multimodal regimens. MN is frequently combined with surgical subcision, chemical peels, and transdermal agents. Platelet‐Rich Plasma (PRP) is the most widely utilized adjunct, while topical human insulin is emerging as a promising alternative.

**Conclusions:**

The mapped evidence supports MN as a safe bridging intervention for multimodal scar management. However, this review identifies a critical methodological gap: the pervasive reliance on truncated follow‐up periods (1 to 3 months). This abbreviated timeline predominantly captures transient inflammatory edema rather than definitive neocollagenesis, risking an overestimation of long‐term efficacy. Future clinical trials must implement a minimum follow‐up of 6 to 12 months alongside objective biometric assessments.

## 1 Introduction

Acne vulgaris often leads to structural deformities known as atrophic acne scars. The pathogenesis involves an altered wound healing response to follicular inflammation, causing collagen and elastin destruction and subsequent dermal volume loss [1, 2]. Using Jacob's classification system, clinicians categorize these structural deficits into three morphological subtypes: rolling, boxcar, and icepick scars [3, 4]. Atrophic scars impose a psychosocial burden, leading to lower self‐esteem and impaired quality of life for patients [5, 6]. Finding effective and safe scar revision modalities remains a priority in dermatology.

Historically, scar resurfacing relied on ablative procedures such as deep chemical peels and fractional lasers. While effective, these devices vaporize the epidermis, causing longer recovery times and increasing the risk of complications, such as Post‐Inflammatory Hyperpigmentation (PIH) in patients with darker skin tones [7]. To address these limitations, Desmond Fernandes introduced percutaneous collagen induction, or microneedling (MN) [8]. This technique uses a device with fine protruding needles to puncture the skin, initiating a regenerative cascade without removing the epidermal layer [8].

The mechanism of action of MN relies on the creation of transient micro‐channels through the stratum corneum into the dermis. This controlled trauma stimulates the natural regenerative mechanisms of the skin while bypassing the severe inflammatory response typical of ablative procedures [2]. The physiological cascade unfolds in three phases: an initial phase involving platelet activation and the release of growth factors [8], a proliferative phase marked by neo‐vascularization, and a remodeling phase where fibroblasts deposit new types I and III collagen [8, 9]. Over the past decades, this technology has evolved from manual dermarollers to motorized pens with adjustable needle penetration depths [1, 7]. The micro‐channels created during the procedure also provide a route for the transdermal delivery of biological agents, enhancing tissue regeneration [10, 11].

Despite the widespread use of MN, the literature remains methodologically heterogeneous. A variety of device types, needle lengths, combination therapies, and clinical evaluation scales are reported [12, 13]. Given this variation, a scoping review is warranted. Following JBI and PRISMA‐ScR guidance, this review maps the protocols, technical parameters, and combination therapies used in mechanical MN for atrophic acne scars in adolescent and adult patients [14].

## 2 Materials and Methods

This scoping review followed the methodological guidance for scoping reviews developed by the JBI [14]. The reporting structure adheres to the PRISMA‐ScR guidelines [15]. Following JBI recommendations, the primary objective is exploratory and descriptive: to identify, map, and report the characteristics of the literature, rather than to evaluate the comparative clinical effectiveness of the interventions. Consequently, a formal risk of bias assessment of the included evidence was not performed [14]. This scoping review was not prospectively registered; scoping reviews are not currently eligible for registration on PROSPERO.

The review question guiding this work was: what is the extent, nature, and state‐of‐the‐art of the available literature regarding the use of mechanical MN in the treatment of acne scars in adult and adolescent patients?

Inclusion and exclusion criteria were established based on the Population, Concept, and Context (PCC) framework:

- Population: The review included studies involving human participants of any age (encompassing adolescents and adults) presenting with scars resulting from acne vulgaris. Studies focused exclusively on active inflammatory acne without reporting scarring outcomes, as well as research on animal or in vitro models, were excluded. Studies investigating other dermatological scar types were excluded unless post‐acne scarring was explicitly analyzed.
- Concept: The core concept was the application of mechanical MN, encompassing manual dermarollers and motorized pens. The literature was mapped regardless of whether MN was investigated as a monotherapy or utilized in combination with other procedural modalities or transdermal products. To maintain the specific focus of this review, treatments utilizing solely energy‐based devices were excluded. However, “hybrid” articles—defined as studies providing data on mechanical MN used in association with fractional microneedling radiofrequency (RFMN) or directly comparing the two treatments—were included. For these hybrid studies, data extraction was strictly limited to the mechanical MN parameters and outcomes.
- Context: No geographical, sociocultural, or clinical setting limitations were imposed.

A broad spectrum of study designs was considered eligible, encompassing primary clinical research (randomized controlled trials, observational studies, split‐face designs, case series, and case reports) and secondary evidence syntheses (systematic reviews, meta‐analyses, and umbrella reviews). In accordance with the JBI methodological guidance for scoping reviews, the inclusion of diverse evidence sources, including narrative reviews, brief communications, and expert opinions, is necessary to comprehensively map the breadth of the available literature. Including primary research of any sample size and expert communications allows for the identification of current technical parameters and emerging protocols. The search was restricted to full‐text articles published in the English language over the last 10 years, a filter applied directly through the PubMed and Scopus database interfaces. This temporal restriction was applied to capture the recent technological evolution of MN devices (from manual rollers to automated motorized pens) and to reflect the most current clinical protocols, including contemporary multimodal approaches with transdermal biological agents. The restriction to English‐language publications reflects the linguistic expertise and translation resources available to the review team; this constraint is acknowledged as a limitation in Section 6.

A systematic literature search was executed across two electronic databases: PubMed and Scopus, covering the period from 05/06/2026 to 10/06/2026, with the final search conducted on 10/06/2026. The search strategy utilized the following string: (“acne scar*” OR “post‐acne scar*” OR “post acne scar*”) AND (microneedling OR “micro needling” OR microneedle* OR “skin needling” OR “needle roller*”). PubMed and Scopus were selected because they are among the largest and most comprehensive biomedical and multidisciplinary databases, and their combined use is a common and accepted approach for scoping reviews conducted with limited resources. The initial search identified a total of 517 records (PubMed = 210, Scopus = 307). Duplicate removal and full‐text retrieval were managed using Zotero (Corporation for Digital Scholarship), which was also used to generate the manuscript bibliography. After the removal of 201 duplicates, 316 unique records were screened by title and abstract by two independent reviewers. During this phase, 140 records were excluded. Of the 176 reports sought for retrieval, 51 were not retrieved. The remaining 125 reports were assessed for full‐text eligibility. Following the full‐text assessment, 40 articles were excluded for the following reasons: language other than English (*n* = 1) and wrong intervention (standalone RFMN/energy‐based devices; *n* = 39). Ultimately, 85 studies met all inclusion criteria and were included in the scoping review. Discrepancies were resolved through discussion and consensus. The complete selection process is visually documented in the appended PRISMA‐ScR flow diagram (Figure 1).

**FIGURE 1:**
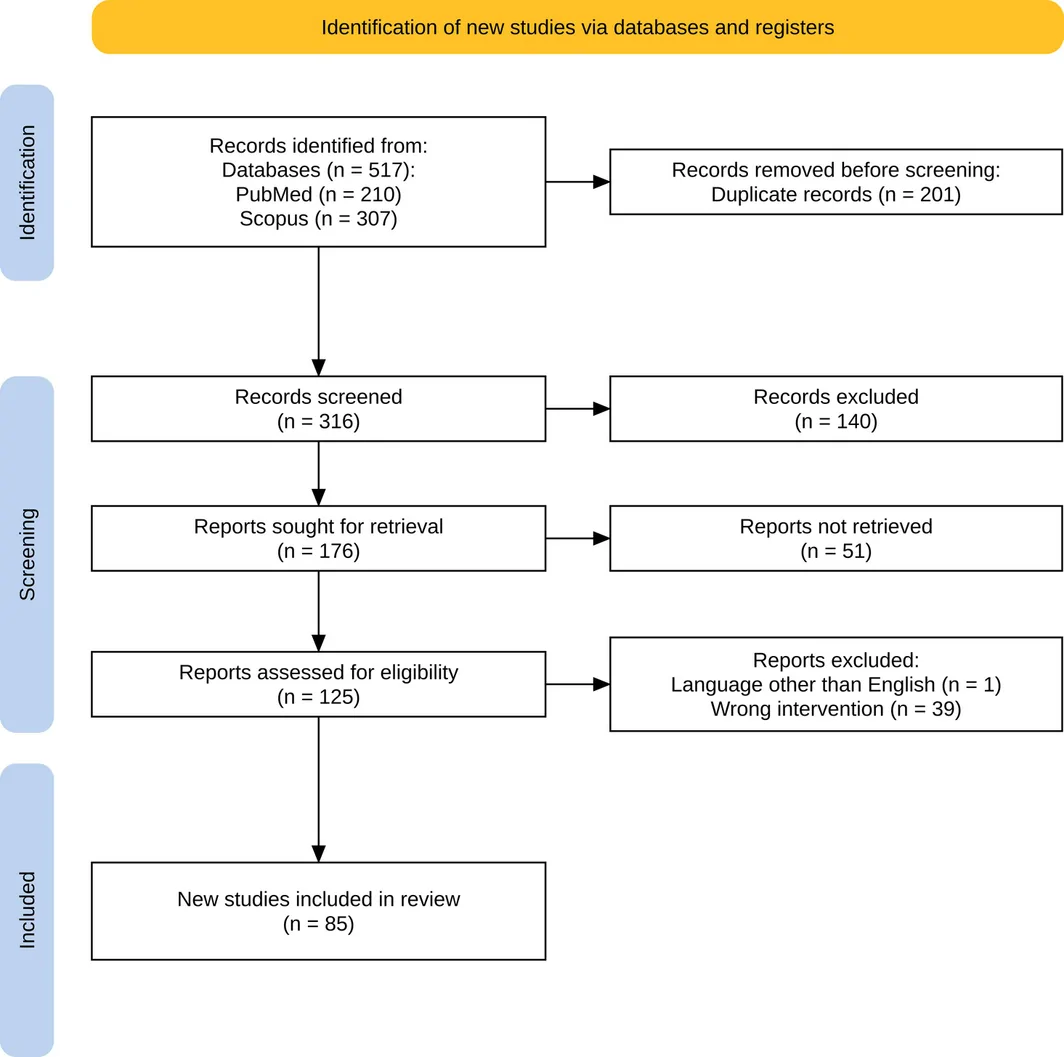
Flow diagram of the literature search and study selection process (*N* = 85).

Data were extracted utilizing a standardized charting form, capturing variables such as author details, publication year, geographical location, study design, demographic characteristics, scar morphology, device specifications, frequency of sessions, combination therapies, and clinical evaluation scales (Table S1). Specifically, this extraction table details the 124 studies that effectively underwent full‐text evaluation; one of the 125 records initially retrieved for full‐text screening was excluded prior to data extraction because, despite presenting an English abstract, the full manuscript was published in a non‐English language. The complete list of the 85 included studies, alongside their detailed extracted variables, is provided in the Table S1. To maintain textual clarity, the main manuscript bibliography reflects only the subset of literature explicitly discussed and referenced within the Introduction, Results, and Discussion sections.

Because this scoping review included both primary clinical studies and secondary evidence syntheses, the review team explicitly acknowledged the risk of “double‐counting” patient cohorts [16]. To mitigate this bias, demographic data and procedural parameters were extracted exclusively from the primary research studies, while secondary evidence syntheses were retained to map overarching conclusions and knowledge gaps. The synthesized data were analyzed using descriptive numerical analysis (frequency counts and proportions) and presented through visual representations. Furthermore, basic qualitative content analysis was employed to categorize the procedural protocols and combination strategies.

## 3 Results

### 3.1 General Characteristics of the Included Studies

A total of 85 studies were included in this scoping review. As illustrated in the geographic distribution map (Figure 2), the mapped literature shows a clear research focus on regions with predominantly darker skin phototypes. Specifically, 81 studies were assigned to single originating countries: India contributed the highest number (*n* = 18), followed by Egypt (*n* = 13), Pakistan (*n* = 13), and China (*n* = 5). Additional contributions originated from the United States (*n* = 5), Iran (*n* = 4), Poland (*n* = 4), Italy (*n* = 3), Taiwan (*n* = 2), Brazil (*n* = 2), and Indonesia (*n* = 2). Ten other countries contributed one study each. To account for the total 85 included studies, the remaining four publications comprise three global multicentric reviews that aggregated data from multiple nations and one clinical trial where the geographic location was not explicitly reported.

**FIGURE 2:**
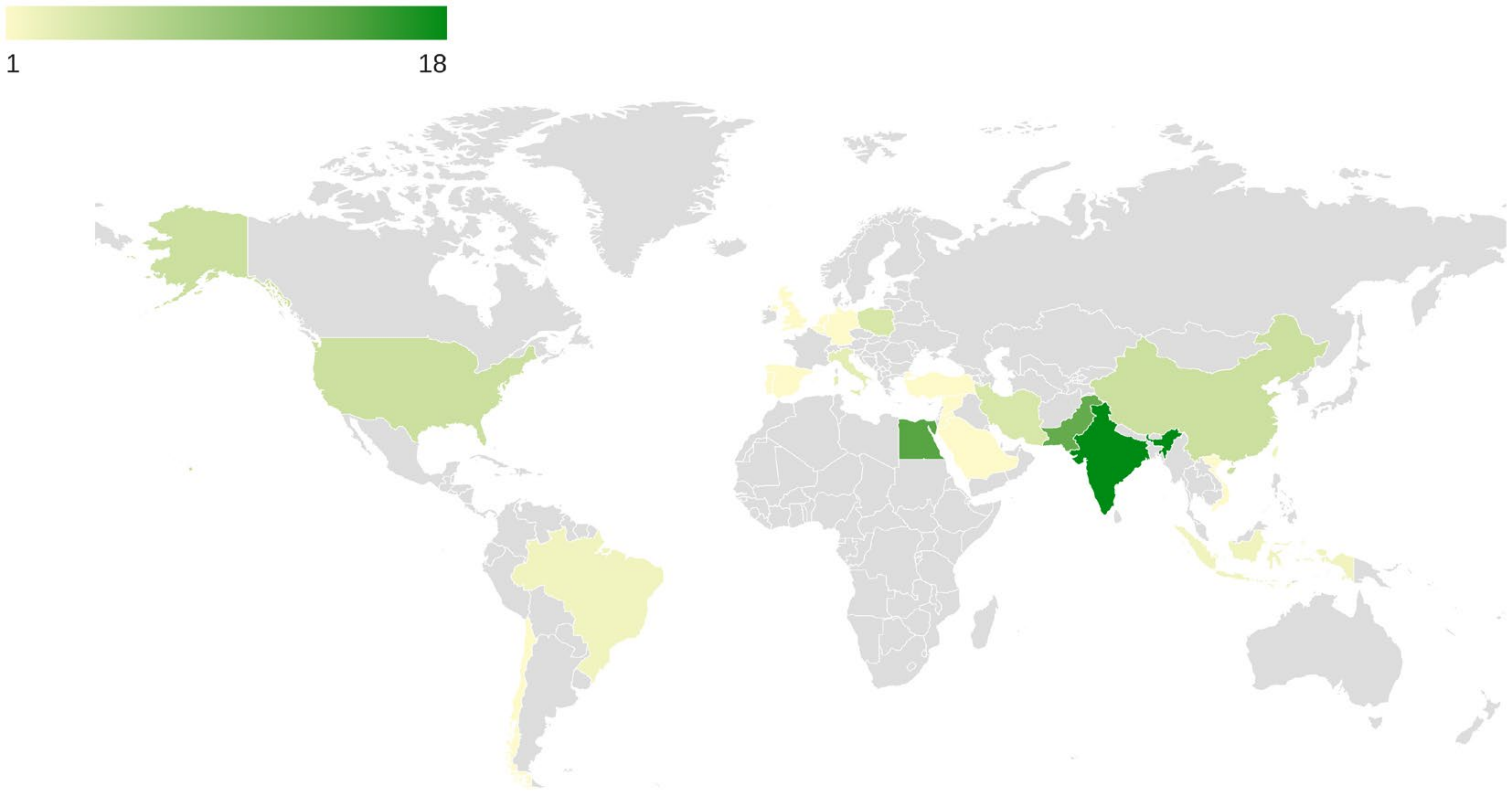
Geographic distribution of country‐specific studies. The overall total includes an additional 3 global multicentric studies that could not be assigned to a single country.

As shown in Figure 3, the included articles cover a range of study designs. We identified 28 evidence syntheses. The remaining 57 primary clinical studies mainly used comparative designs (*n* = 41). This group includes Randomized Controlled Trials (RCTs) with parallel or split‐face frameworks, along with non‐randomized quasi‐experimental studies. Finally, the review includes 13 single‐arm observational studies and 3 case reports or series.

**FIGURE 3:**
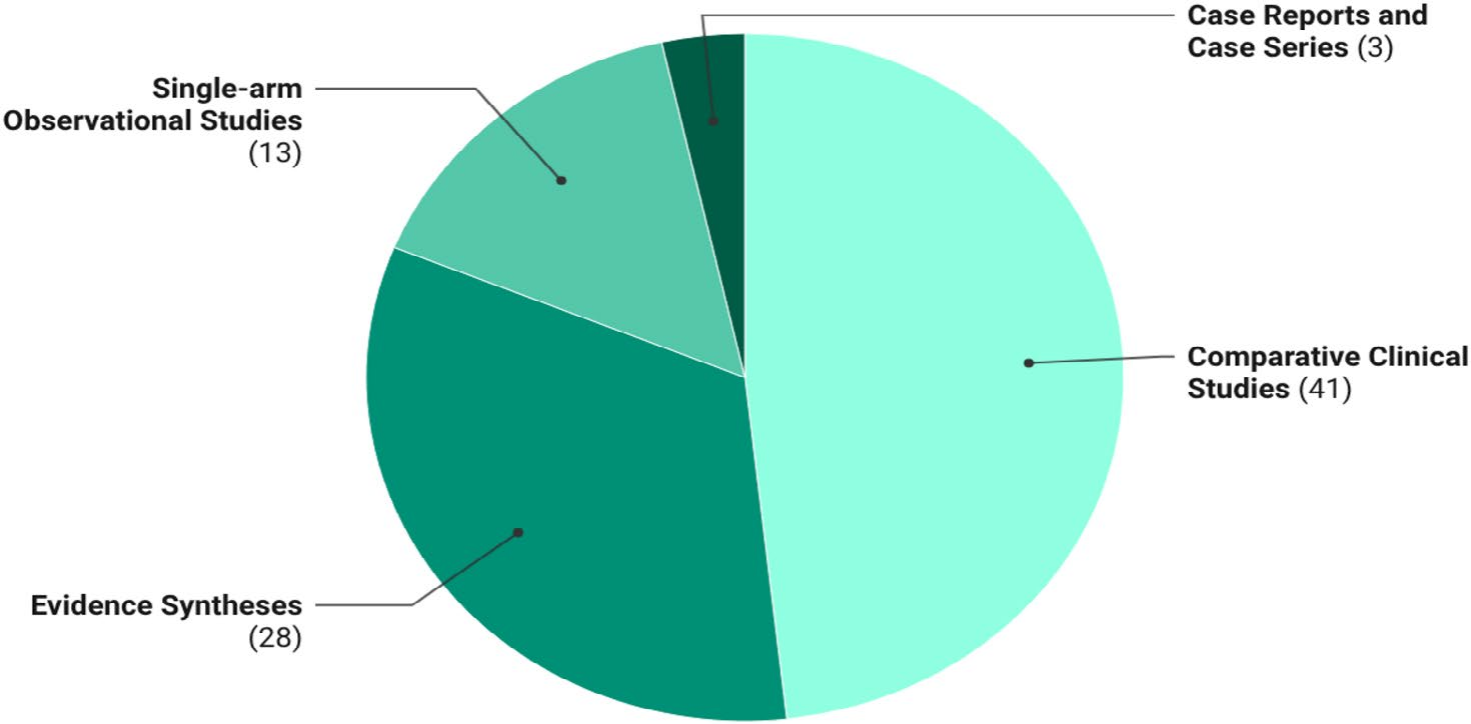
Proportional breakdown of the methodological designs used across the analyzed publications (*N* = 85).

Demographic data extracted from primary studies reveal sample sizes ranging from single cases to 139 patients [17]. Mean ages ranged from 23 to 32 years, with a generally slight female predominance. A subset of trials omitted basic demographic details, such as exact mean age or gender distribution [18]. The majority of studies evaluated cohorts with mixed presentations of atrophic scar subtypes, using frameworks such as Jacob's classification to categorize lesions into rolling, boxcar, and icepick scars [19, 20].

Clinical outcomes across the full set of included studies (both primary research and secondary evidence syntheses) were quantified using a specific array of tools, as summarized in Figure 4. The Goodman and Baron grading system (qualitative and quantitative) remained the standard framework, used in 58 studies. Subjective clinical improvement and patient satisfaction were frequently assessed using Quartile Grading Scales and Likert questionnaires (*n* = 32). Procedural pain and tolerability were monitored using the Visual Analog Scale (VAS) in 19 trials. The Échelle d'Évaluation Clinique des Cicatrices d'Acné (ECCA) scale was mapped in 11 studies. Objective physical assessments, including advanced 3D multispectral imaging, ultrasound, and histological biopsies, were used in 14 studies to provide non‐invasive and cellular‐level data. Finally, the psychosocial impact of scar revision was evaluated using Quality of Life metrics, such as the Dermatology Life Quality Index (DLQI) and the Cardiff Acne Disability Index (CADI), in 8 studies.

**FIGURE 4:**
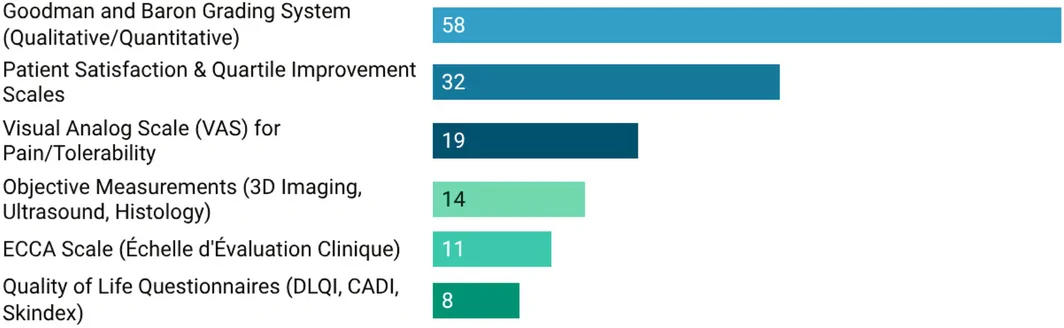
Clinical outcome measures and assessment tools. Values represent the absolute number of studies employing each specific method. Multiple choices per study apply.

### 3.2 Mechanical MN Monotherapy Protocols

Extracted data show the use of both manual dermarollers and automated motorized pens, with manual devices typically fitted with needles ranging from 1.0 to 2.0 mm in length [21]. The standard protocol involved rolling the device over stretched skin in multiple directions (vertical, horizontal, and both diagonals) for several passes [22].

Studies using automated motorized pens reported dynamic adjustments of needle penetration depth during the procedure, typically ranging from 0.5 to 3.0 mm. Cartridge configurations varied across the literature, including 12‐needle and 36‐needle setups. Across all primary trials using mechanical MN, regardless of device type, the clinical endpoint was consistently the appearance of uniform pinpoint bleeding.

### 3.3 Multimodal and Combination Protocols

The majority of the mapped primary research investigated the use of MN in combination with other therapeutic modalities. Instrumental combinations included surgical subcision performed prior to the MN procedure to sever fibrous bands. Subcision protocols varied from the use of traditional hypodermic needles to blunt cannulas to mitigate bleeding [17, 23, 24]. Furthermore, MN was mapped as an adjuvant to energy‐based resurfacing, such as fractional carbon dioxide or Erbium lasers.

Studies extensively reported using the MN device to facilitate the transdermal delivery of biological agents. Autologous Platelet‐Rich Plasma (PRP) was the most frequently mapped adjunct, applied topically or injected intradermally [22, 25]. Topical human insulin was reported as an alternative transdermal agent, utilized at concentrations of 40 IU/mL or 100 IU/mL [26, 27]. Additional agents mapped in the literature included non‐cross‐linked hyaluronic acid, epidermal growth factors, botulinum toxin A, and amniotic fluid‐derived mesenchymal stem cell products [28, 29, 30].

Finally, procedural combinations included the concurrent application of chemical peels, using formulations such as glycolic acid and trichloroacetic acid (TCA) [31]. For severe scar morphologies, researchers mapped the Chemical Reconstruction of Skin Scars (CROSS) technique, which involves the focal application of high‐concentration acids directly into the scar depressions prior to full‐field MN [7, 17].

## 4 Discussion

Atrophic acne scars present a structurally heterogeneous challenge. Traditional resurfacing modalities, such as fractional lasers and deep chemical peels, provide dermal remodeling but entail prolonged epidermal recovery and a risk of PIH, particularly in patients with Fitzpatrick skin phototypes III–VI [32, 33]. MN bypasses these limitations by creating transient vertical micro‐channels that trigger a regenerative neocollagenesis cascade while preserving the stratum corneum [1, 8]. This mechanism explains the demographic distribution of the mapped literature: the majority of clinical trials originate from regions with predominantly darker skin phototypes, reflecting the clinical necessity for a non‐thermal alternative to ablative lasers in these populations. Consequently, MN demonstrates a consistent safety profile across mapped devices, characterized by a short downtime of 1 to 3 days and a low incidence of dyspigmentation [34, 35].

This scoping review synthesized the technical parameters of the utilized devices. The extracted data highlight a dichotomy in device technology, transitioning from manual dermarollers (typically equipped with 192 needles) to automated motorized pens (predominantly 36‐needle cartridges) [28, 36]. Standard penetration depths of 1.5 to 2.0 mm are used to target the superficial and mid‐dermis. The clinical shift toward automated pens is driven by their capacity for dynamic depth adjustments (up to 3.0 mm), which allows practitioners to tailor penetration according to regional skin thicknesses across the face [21]. Furthermore, automated vertical stamping devices reduce operator‐dependent variables and mitigate the risk of “tram‐track” hypertrophic scarring occasionally associated with the angled penetration trajectory of manual rollers [9, 34]. Despite this technological transition, there remains a critical gap in the literature: the absence of head‐to‐head randomized clinical trials directly comparing the efficacy of manual versus motorized devices.

The mapped evidence also indicates a biomechanical limitation: MN monotherapy yields suboptimal efficacy for complex atrophic lesions, particularly narrow icepick scars and deeply anchored rolling scars. This discrepancy relates to the physical interaction between the needle and the scar architecture [37, 38]. To address subdermal adhesions, surgical subcision is a prerequisite for severe rolling scars to physically release the fibrous bands anchored to the deep dermis and dermal‐fat junction [23, 39]. For icepick lesions, the focal chemical ablation of scar shoulders via the CROSS technique is frequently employed prior to full‐field MN [7, 17]. In these multimodal protocols, mechanical MN acts as a bridging intervention.

The procedure bypasses the stratum corneum, facilitating the immediate transdermal delivery of biological agents into the dermis. Autologous Platelet‐Rich Plasma (PRP) is the most frequently mapped adjunct, applied sequentially after the mechanical trauma to accelerate tissue remodeling. However, the literature reveals a lack of standardization in PRP preparation protocols, centrifugation speeds, and application methods, compromising cross‐study reproducibility [10, 40]. This methodological inconsistency explains the increase in trials exploring topical human insulin as a standardized and cost‐effective alternative to PRP [26, 27]. Additionally, studies report the transdermal delivery of botulinum toxin A to paralyze superficial muscle fibers, aiming to eliminate the dynamic pulling effect on the scar bed. The clinical utility of this approach remains debated due to the transient nature of muscular paralysis, which may lead to a recurrence of scar tethering once the neurotoxin effect subsides [41]. While the clinical application of MN is documented, this review identifies methodological limitations that impede the establishment of definitive guidelines. First, the cited clinical endpoint of “pinpoint bleeding” remains a visual, operator‐dependent marker lacking objective quantification. Second, clinical improvements are primarily assessed using the qualitative Goodman and Baron scale, which introduces observer bias. However, a shift is emerging in recent literature, moving from subjective visual grading to objective biometrics. Studies have adopted 3D multispectral imaging (e.g., VISIA) to objectively quantify scar severity and porphyrin levels [28]. Advanced ultrasound technologies, such as Ultrasound Shear Wave Elastography (SWE) and high‐frequency color Doppler ultrasound, evaluate the biomechanical elasticity of the newly formed collagen and measure scar depth [42, 43].

Finally, a pervasive methodological flaw across the mapped literature is the reliance on truncated follow‐up periods. The vast majority of clinical trials assess final outcomes between one and three months after the last treatment session [31]. This abbreviated timeline is frequently adopted in clinical trial designs to maximize patient compliance, minimize dropout rates, and rapidly publish results based on early aesthetic improvements. However, from a histological and biomechanical perspective, this timeframe is fundamentally inadequate. The physiological cascade of percutaneous collagen induction and deep dermal remodeling is a slow process. While initial fibroblastic activity may be observed at 6 weeks, complete neocollagenesis and continued improvement in skin texture require a maturation period of 6 to 12 months [1, 8]. Consequently, these early evaluations primarily capture transient inflammatory edema and early extracellular matrix deposition. This early assessment risks overestimating the immediate efficacy of the intervention while failing to document the definitive stabilization of the dermal architecture, potentially masking long‐term scar relapse once the post‐procedural swelling completely subsides.

## 5 Conclusions

MN has progressively evolved from an isolated collagen induction technique into a central platform of multimodal acne scar therapy. To our knowledge, this is the first scoping review specifically mapping the procedural characteristics of mechanical MN, including device configuration, penetration depth, procedural endpoints, and multimodal integration. This focus contrasts with prior reviews that concentrated exclusively on comparative clinical outcomes. Based on the mapped evidence, this scoping review posits that MN monotherapy often presents biomechanical limitations when treating complex atrophic acne scarring. Because vertical mechanical needles cannot shear deep horizontal fibrosis or completely reach narrow epithelial tracts, the procedure is increasingly used as a critical bridging mechanism. It facilitates the transdermal delivery of biological agents and evens out superficial skin texture within a broader surgical and chemical sequence.

While the safety profile of MN is highly favorable compared to ablative lasers for patients with darker skin phototypes, the current literature presents notable methodological shortcomings. Clinical efficacy may be overestimated due to the pervasive use of truncated follow‐up periods. Evaluating patients at 1 to 3 months post‐treatment predominantly captures transient inflammatory edema rather than the definitive maturation of neocollagenesis. Therefore, future clinical trials should strongly consider a minimum follow‐up of 6 to 12 months to accurately document true dermal remodeling and monitor potential post‐inflammatory scar relapse.

Furthermore, the lack of standardization in adjuvant therapies limits cross‐study reproducibility. The considerable variability of PRP preparation protocols currently challenges the establishment of universal clinical guidelines. In this context, the emerging application of topical human insulin represents a promising, standardized, and cost‐effective alternative that warrants rigorous, large‐scale clinical exploration to overcome some of the practical limitations of blood‐derived products.

Ultimately, to elevate the scientific rigor of MN research, clinical evaluations should progressively move beyond subjective visual grading scales. The clinical validation of dynamic automated devices and novel multimodal protocols would be significantly enhanced by the broader adoption of objective biometric technologies, such as multispectral 3D imaging and ultrasound elastography. This shift can advance the evaluation of acne scar revision from visual estimation to precise morphological quantification.

## 6 Limitations

This review has several methodological limitations. Consistent with the exploratory framework of the JBI guidelines, a formal risk of bias or methodological quality assessment of the included literature was not conducted. To fulfill the primary objective of comprehensively mapping the state‐of‐the‐art of MN protocols, this review aggregated a broad spectrum of study designs, from randomized controlled trials to case reports, narrative reviews, and expert opinions. This inclusive approach maximized data comprehensiveness but compromised the methodological homogeneity of the synthesized evidence. The search was also restricted to two databases (PubMed and Scopus), excluding gray literature, institutional repositories, and unpublished trial registries. Finally, the 10‐year temporal filter and English‐language restriction may have introduced selection bias, excluding relevant historical data and international protocols published in other languages.

## Funding

The authors have nothing to report.

## Ethics Statement

The authors have nothing to report.

## Conflicts of Interest

The authors declare no conflicts of interest.

## Supporting information


**Table S1:** Data extraction table of all studies assessed at the full‐text stage of the scoping review on microneedling for the treatment of acne scars (both included and excluded studies), detailing author and year, country, study design, sample size, sex distribution, mean age, scar type, device type, technical parameters, treatment frequency and number of sessions, comparator/combination therapies, clinical evaluation scales used, and follow‐up time.

## Data Availability

The data that supports the findings of this study are available in the Supporting Information of this article.
